# Silent subepicardial hematoma due to spontaneous coronary artery rupture in a patient with Graves’ disease

**DOI:** 10.1186/s44215-023-00106-6

**Published:** 2023-09-05

**Authors:** Kanji Matsuzaki, Kisato Mitomi, Akito Imai, Yasunori Watanabe

**Affiliations:** 1 Department of Cardiovascular Surgery, Hitachi General Hospital, 2-1-1 Jonan, Hitachi, Ibaraki 317-0077 Japan; 2 University of Tsukuba Hospital, 2-1-1 Amakubo, Tsukuba, Ibaraki 305-8576 Japan

**Keywords:** Subepicardial hematoma, Spontaneous coronary artery rupture, Proximal right coronary artery

## Abstract

**Background:**

Subepicardial hematoma due to spontaneous coronary artery rupture (SCAR) is exceedingly rare.

**Case presentation:**

An asymptomatic 68-year-old woman with Graves’ disease was introduced to us because of an abnormal right hilar shadow. Graves’ disease had been treated effectively with thiamazole. Without any chest trauma or disease related to coronary artery rupture, computed tomography revealed a 44-mm mass lesion that occupied the front of the right atrium. Two branches arising from the proximal right coronary artery surrounded the lesion and appeared to be the feeding vessels. We suspected a tumor and performed radical surgery. The lesion was completely removed using an ultrasonic scalpel and revealed as organized subepicardial hematoma.

**Conclusion:**

This is the rare surgical case of organized subepicardial hematoma due to SCAR. A high grade of suspicion is necessary for the correct diagnosis. Graves’ disease is not considered as an underlying disease of SCAR.

## Background

Subepicardial hematoma is rare. It is mostly caused by blunt chest trauma [1], catheter cardiac intervention [2], cardiac surgery, or acute myocardial infarction. Coronary artery rupture without any coronary intervention, known as spontaneous coronary artery rupture (SCAR), is also rare [3]. Its major manifestation is cardiac tamponade that may develop cardiogenic shock [4]. Subepicardial hematoma due to SCAR is exceedingly rare. Its etiology is atherosclerotic disease, aneurysm, trauma, infection [5], dissection, Kawasaki’s disease, Bechet’s disease [6], or Ehlers-Danlos syndrome [7]. To the best of our knowledge, there is no report of Graves’ disease as an underlying disease.

## Case presentation

A 68-year-old woman was introduced to us because an abnormal shadow of the right lung hilar region was found on chest X-ray at her periodical health examination. The patient had undergone abdominal hysterectomy for uterine myoma at the age of 50 years old and had been treated with thiamazole for Graves’ disease in the previous 6 months before visiting our hospital. She was asymptomatic and had no history of blunt chest trauma, cardiac intervention, myocardial infarction, or known disease related to SCAR.

**Fig. 1:**
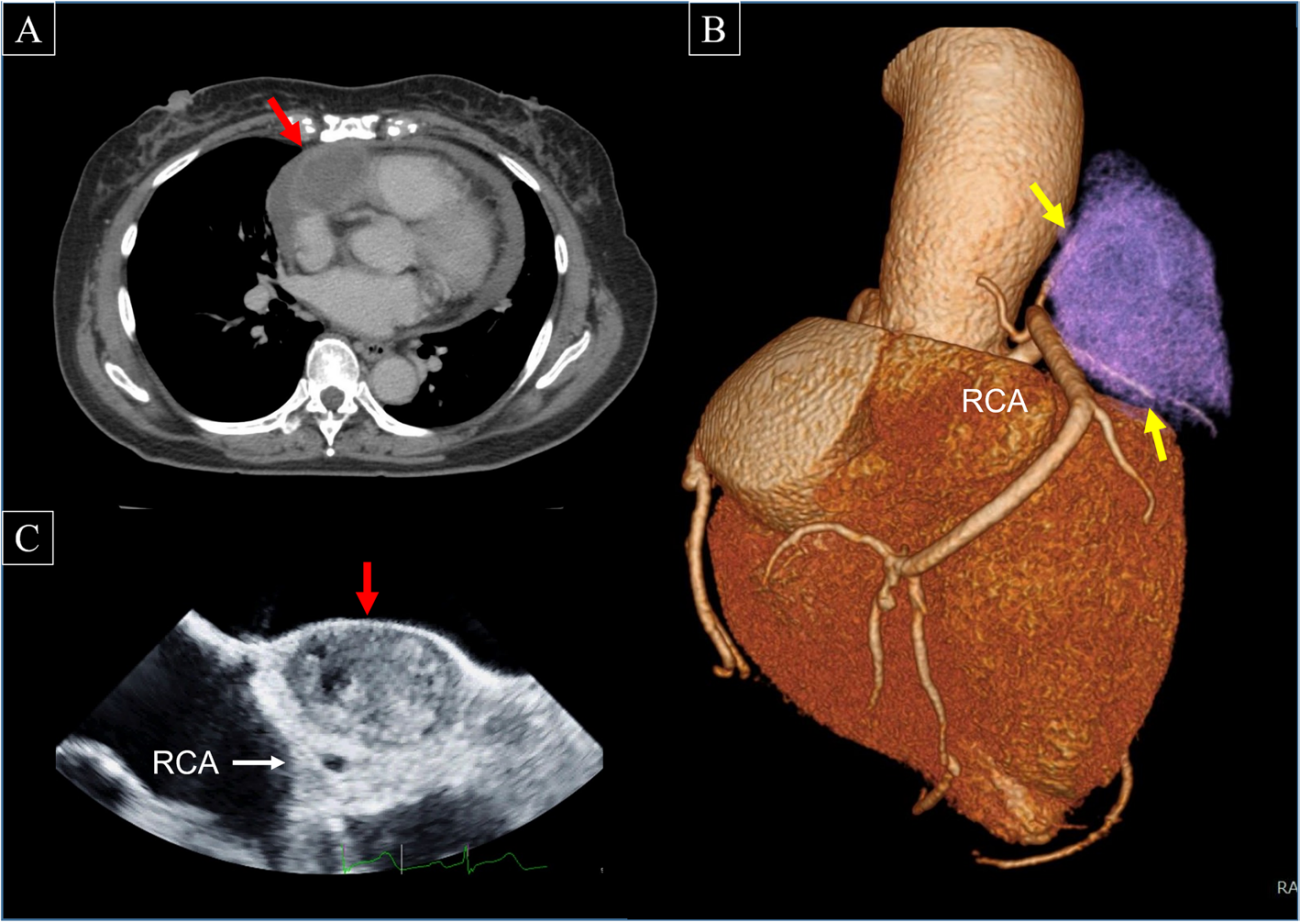
**A** Computed tomography revealing a 44-mm mass lesion (red arrow) occupying the front of the right atrium. **B** Multidetector computed tomography revealing two branches of the proximal right coronary artery surrounding the lesion (yellow arrows). **C** Epicardial direct echocardiography demonstrating an oval mass with high echoic matrix (red arrow)

Computed tomography revealed a 44-mm mass lesion that occupied the front of the right atrium, which was accompanied with pericardial effusion (Fig. 1A). There was no coronary aneurysm, dissection, or stenosis. Two branches arising from the proximal right coronary artery (RCA), the conus branch and one of the right ventricular branches, surrounded the lesion and appeared to be the feeding vessels (Fig. 1B). We first suspected a tumor. There was neither significant enlargement of the mediastinal lymph node nor abnormal lesion suggesting metastasis. Her blood tests showed an increased thyroid-stimulating hormone level of 5.85 µIU/mL (reference 0.27 ~ 4.2), a normal free-T3 level of 2.75 pg/mL (reference 2.3 ~ 4.0) and a decreased free-T4 level of 0.61 ng/dL (reference 1.0 ~ 1.8). Other physical examinations and laboratory tests were unremarkable.

**Fig. 2:**
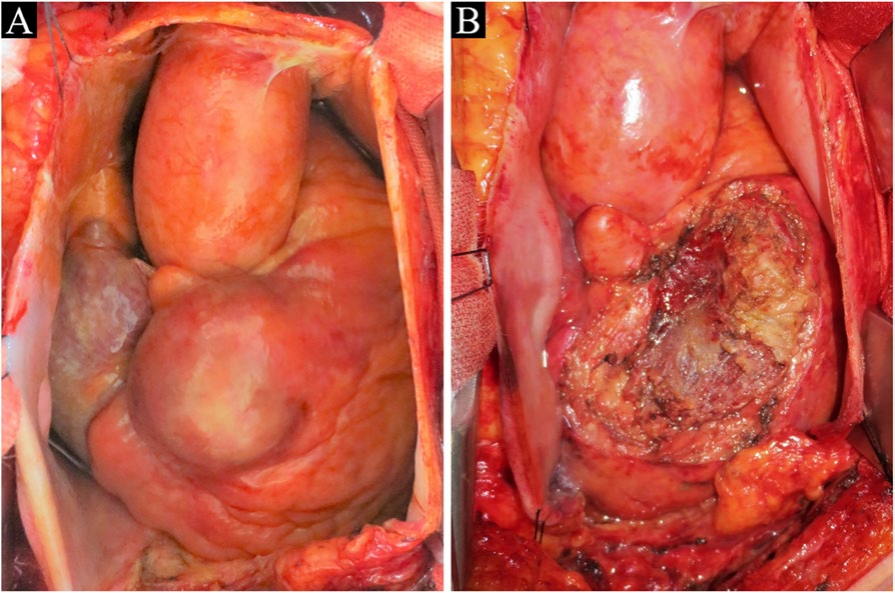
**A** A 5-cm mass on the right ventricular outflow tract. **B** No major injury over the right ventricular myocardium after removal of the lesion

**Fig. 3:**
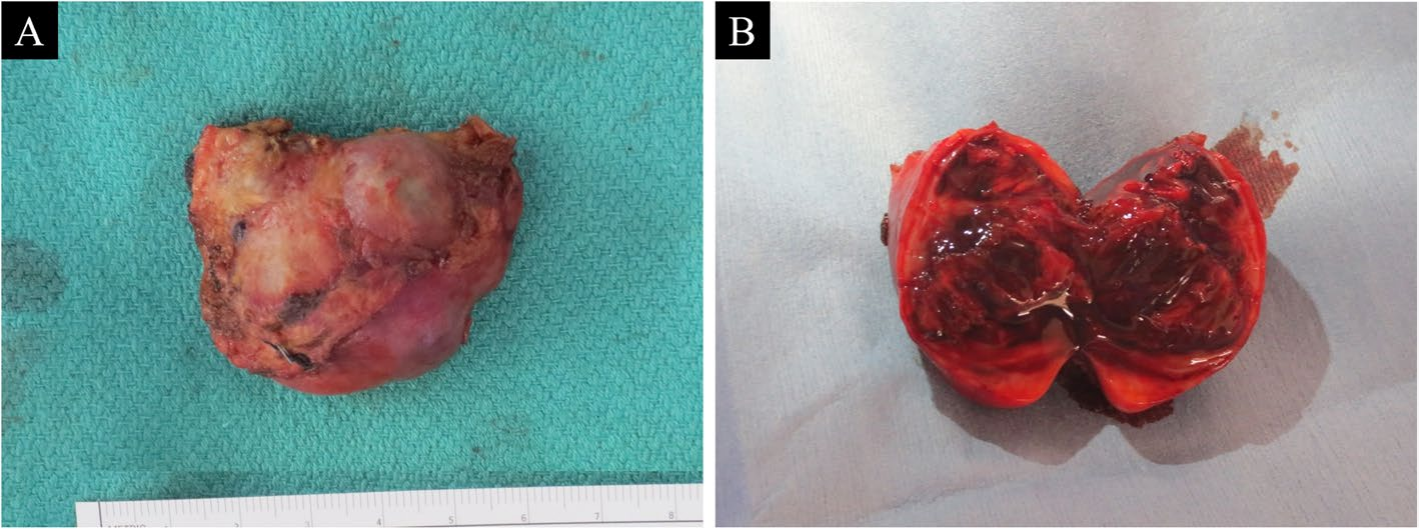
**A** The lesion removed in one block. **B** Organized hematoma containing some blood clots

We performed an elective surgery. After median sternotomy, there was no abnormality at the anterior mediastinum and over the pericardium. Pericardiotomy revealed a 5-cm mass riding on the right ventricular outflow tract (Fig. 2A) and pericardial effusion containing old blood. The lesion had a smooth round surface, dark red appearance, and elastic firm touch. Epicardial direct echocardiography demonstrated an oval mass with high echoic matrix and the RCA passing beneath that (Fig. 1C). Intraoperative rapid pathological examination of the sample tissue revealed no significant finding suggesting malignancy. Without cardiopulmonary bypass, the entire lesion was carefully dissected from the right ventricular myocardium using an ultrasonic scalpel. The accompanying branches from the proximal RCA were ligated, and the lesion was successfully removed in one block (Fig. 3A). There was no major injury over the coronary artery or myocardium around the right ventricular outflow tract (Fig. 2B).

The specimen was 45 × 30 × 20 mm in size, and the pathological diagnosis was an organized hematoma containing some blood clots (Fig. 3B). The accompanying vessel had a histological structure consistent with a coronary artery branch. The patient restarted medicine for Graves’ disease and recovered well without any complications. Since then, she has been living a normal life without recurrence for more than 3 years.

## Discussion and conclusions

The present patient was treated effectively with thiamazole for Graves’ disease. To the best of our knowledge, this disease has not been reported as an underlying disease of SCAR. Thiamazole, also known as methimazole in the USA, is an antithyroid medicine that may induce antineutrophil cytoplasmic antibody-associated vasculitis [8]. It is an autoimmune disorder affecting small to medium-sized vessels, and usually causes rapidly progressive glomerulonephritis (hematuria, proteinuria), pulmonary hemorrhage (cough, hemoptysis, dyspnea), fever, arthralgia, arthrophyma, skin ulcer, or purpura [9]; however, the patient did not present such symptoms. According to a previous study, its cardiac involvement is minor, and its common cardiac manifestations are pericarditis and cardiomyopathy [10]. There is a case report of angina pectoris but no report of SCAR as a coronary manifestation [11]. In the present case, Graves’ disease was not thought to be the etiology of her subepicardial hematoma.

We first suspected that the mass lesion was a tumor. Hematoma was not assumed because the patient had no symptom nor history that suggested vascular events. Preoperative computed tomography and echocardiography also did not suggest subepicardial hematoma. A high grade of suspicion was necessary for making the correct diagnosis. Magnetic resonance imaging should have been performed before surgery. Although needle biopsy was recommended for diagnosis, it was too dangerous as the mass was beside the beating heart. Since the possibility of malignancy could not be ruled out, we decided to perform radical surgery. If the correct diagnosis of organized hematoma were obtained, just follow-up may have been suitable. Fortunately, the hematoma was removed successfully without cardiopulmonary bypass which stood by during surgery. However, we should have used that from the first in the case of contained coronary rupture.

As mentioned previously, subepicardial hematoma due to SCAR is exceedingly rare. Longobardi et al. reported that rupture of the distal RCA or left coronary artery usually manifests as intrapericardial bleeding, and that proximal RCA potentially presents with a contained rupture [4]. The former is a more fatal condition because cardiac tamponade often evolves into cardiogenic shock. On the other hand, the latter is less serious with slower development of circulatory disturbance. The proximal RCA has anatomical advantages for allowing contained rupture such as being well embedded in the anterior atrioventricular groove [4]. In the present case, its onset and following course were undetected and the hematoma had enough time to be organized one.

In the surgery, epicardial direct echocardiography was helpful for detecting the actual location of the RCA behind the hematoma in order to prevent coronary injury. We used a general probe (Philips S5-1 broadband sector array transducer) for the mass lesion and a small L-shaped probe (Philips L15-7io broadband compact linear array transducer) for the RCA. An ultrasonic scalpel was useful for dissecting between the organized hematoma and the right ventricular myocardium. This instrument can safely dissect the epicardium while gently making contact with the coronary artery and myocardium. We could expose the accompanying branches from the proximal RCA, which were ligated successfully. An ultrasonic scalpel was also useful for stopping minor bleeding around the organized hematoma. The hematoma was removed completely from the beating heart without major cardiac injury.

In conclusion, a 68-year-old woman with Graves’ disease developed a silent subepicardial hematoma that mimicked a tumor. This is the rare surgical case of organized subepicardial hematoma due to SCAR of the proximal RCA. A high grade of suspicion is necessary for the correct diagnosis. Graves’ disease is not considered as an underlying disease of SCAR.

## Data Availability

Data sharing is not applicable to this article as no datasets were generated or analyzed during the current study.

## Abbreviations

SCAR: Spontaneous coronary artery rupture
RCA: Right coronary artery
